# Comprehensive analysis of prediction of the EGFR mutation and subtypes based on the spinal metastasis from primary lung adenocarcinoma

**DOI:** 10.3389/fonc.2023.1154327

**Published:** 2023-04-18

**Authors:** Ran Cao, Huanhuan Chen, Huan Wang, Yan Wang, E-Nuo Cui, Wenyan Jiang

**Affiliations:** ^1^ Department of Biomedical Engineering, School of Intelligent Medicine, China Medical University, Liaoning, Shenyang, China; ^2^ Department of Oncology, Shengjing Hospital of China Medical University, Shenyang, China; ^3^ Radiation Oncology Department of Thoracic Cancer, Cancer Hospital of China Medical University, Liaoning Cancer Hospital and Institute, Liaoning, Shenyang, China; ^4^ School of Computer Science and Engineering, Shenyang University, Shenyang, China; ^5^ Department of Scientific Research and Academic, Cancer Hospital of China Medical University, Liaoning Cancer Hospital and Institute, Liaoning, Shenyang, China

**Keywords:** EGFR, spinal metastasis, NSCLC, radiomics, MRI

## Abstract

**Purpose:**

To investigate the use of multiparameter MRI-based radiomics in the in-depth prediction of epidermal growth factor receptor (EGFR) mutation and subtypes based on the spinal metastasis in patients with primary lung adenocarcinoma.

**Methods:**

A primary cohort was conducted with 257 patients who pathologically confirmed spinal bone metastasis from the first center between Feb. 2016 and Oct. 2020. An external cohort was developed with 42 patients from the second center between Apr. 2017 and Jun. 2021. All patients underwent sagittal T1-weighted imaging (T1W) and sagittal fat-suppressed T2-weight imaging (T2FS) MRI imaging. Radiomics features were extracted and selected to build radiomics signatures (RSs). Machine learning classify with 5-fold cross-validation were used to establish radiomics models for predicting the EGFR mutation and subtypes. Clinical characteristics were analyzed with Mann-Whitney U and Chi-Square tests to identify the most important factors. Nomogram models were developed integrating the RSs and important clinical factors.

**Results:**

The RSs derived from T1W showed better performance for predicting the EGFR mutation and subtypes compared with those from T2FS in terms of AUC, accuracy and specificity. The nomogram models integrating RSs from combination of the two MRI sequences and important clinical factors achieved the best prediction capabilities in the training (AUCs, EGFR vs. Exon 19 vs. Exon 21, 0.829 vs. 0.885 vs.0.919), internal validation (AUCs, EGFR vs. Exon 19 vs. Exon 21, 0.760 vs. 0.777 vs.0.811), external validation (AUCs, EGFR vs. Exon 19 vs. Exon 21, 0.780 vs. 0.846 vs.0.818). DCA curves indicated potential clinical values of the radiomics models.

**Conclusions:**

This study indicated potentials of multi-parametric MRI-based radiomics to assess the EGFR mutation and subtypes. The proposed clinical-radiomics nomogram models can be considered as non-invasive tools to assist clinicians in making individual treatment plans.

## Introduction

Lung cancer is one of the most common malignant tumors worldwide, with non-small-cell lung cancer (NSCLC) composes approximately 80% of all cases (1) (2). The most frequent type of lung cancer is lung adenocarcinoma, accounting for about 40% (3). It has been confirmed that the continuous activation of epidermal growth factor receptor (EGFR) tyrosine kinase domain in tumor tissues is caused by the mutation of EGFR in lung adenocarcinoma (4) (5). Therefore, the mutation status of EGFR is the key factor to determine the therapeutic effect of EGFR-tyrosine kinase inhibitors (TKIs) (6). Clinical trials have shown that patients with EGFR mutation often have a longer progression free survival compared with those with wild type EGFR (7). The main mutation sites of the EGFR gene were 18, 19, 20 and 21, and the exons 19/21 are the most common mutations (8) (9). The overall survival time of targeted therapy patients with exon 19/21 mutations is ranging from 41 to 44 months, which is longer than that of patients with exon 18 mutation (19 months) (10). Since the EGFR status lead to different prognosis, determination of specific mutation subtypes plays a guiding role in the subsequent treatment planning.

The spine is a common site of the metastatic spread in NSCLC (11). The spinal metastases may cause erode of normal spinal tissues, forming intratumor lesion regions. Although assessment of the EGFR mutation status can be conducted with the biopsy puncture, an accurate localization of the puncture is often difficult to determine, and has a high false-positive rate (12) (13). Besides, the puncture may cause damages to the nerves and lead to metastasis (14) (15). Therefore, there is an urgent need for a non-invasive method to assess the EGFR mutation status to assist clinicians in making individual treatment plans.

In recent years, radiomics has been an emerging field in the oncology, which can quantitatively describe relationships between imaging features and underlying tumor pathophysiology by extracting and analyzing a large number of quantitative features (16) (17) (18) (19). Previous investigations on evaluating the EGFR mutation status in lung adenocarcinoma mainly focused on primary tumors (20) (21) or brain metastases (22) (23). There are relatively few predictions about EGFR mutation status and mutation subtypes in spinal bone metastasis. A recent study has revealed the association between MRI features derived from bone metastasis and the EGFR mutation sites in exons (24). While, the report enrolled a limited sample size and focused on differentiating the exon 19/21 mutation sites. This study aims to evaluate features from T1W and T2FS MRI and important clinical factors from lung adenocarcinoma patients with spinal metastases, and to develop a radiomics nomogram for prediction of the EGFR mutation and subtypes.

## Materials and methods

### Patients

**Figure 1:**
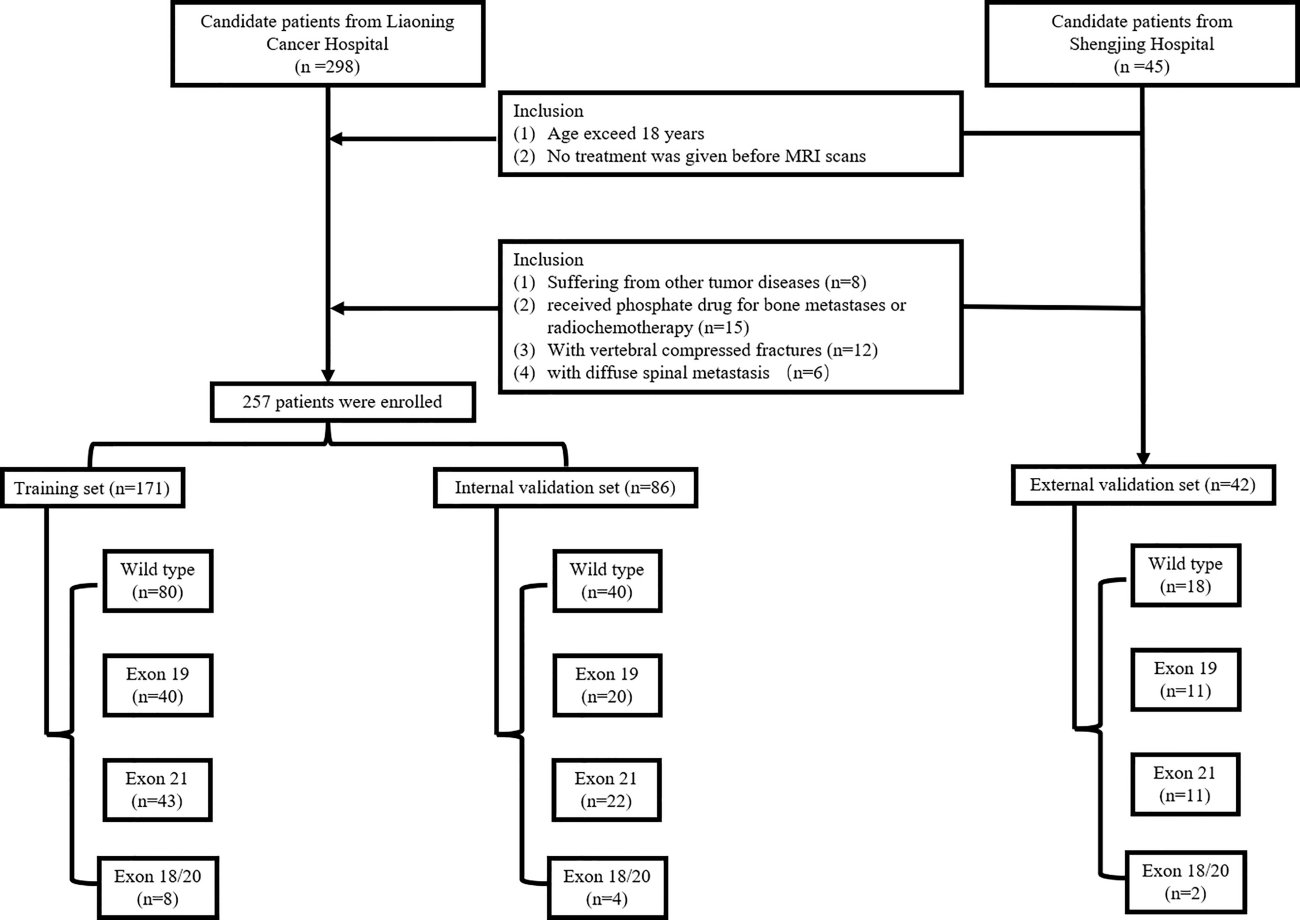
The patient selection and workflow.

A total of 257 patients (mean age: 59.95; ranging from 29 to 89) were included between Feb. 2016 and Oct. 2020 from our hospital (Center 1) and used to build the internal validation set. A total of 42 patients (mean age: 60.12; ranging from 42 to 75) were collected from another hospital (Center 2) between Apr. 2017 and Jun.2021and used as an external validation set. The patients‘ primary lung adenocarcinoma was pathologically diagnosed. The development of bone metastasis were identified by PET/MRI imaging and patient follow-ups. The EGFR mutation status and mutation subtypes were determined by DNA sequencing analysis. Inclusion criteria were: (1) age exceed 18 years and (2) no treatment was given before MRI scans. Exclusion criteria were: (1) suffering from other tumor diseases; (2) received phosphate drug for bone metastases or radiochemotherapy; (3) with vertebral compressed fractures; and (4) with diffuse spinal metastasis. The inclusion flowchart is shown in **Figure 1**. All patients were randomly divided at a 2:1 ratio into training and internal validation sets by stratified sampling. Clinical characteristics included gender, age, smoking, performance status (PS), cytokeratin (CYFRA), serum carcinoembryonic antigen (CEA) level, and neuron specific enolase (NSE). This retrospective study was approved by the institutional review board, and the requirement for informed consent was waived.

### Data acquisition and spinal metastases segmentation

Before surgery, all patients were scanned using a Siemens 3.0T MRI scanner (Siemens magnetic trio, Erlangen, Germany). MRI parameters were: sagittal T1-weighted imaging (repetition time [TR] = 500ms, echo time [TE] = 9ms). Sagittal fat-suppressed T2-weight imaging (TR = 3000 ms, TE = 78 ms), sagittal slice thick-ness 4 mm, scanning interval 4.4 mm; axial slice thickness 4.5 mm, scanning interval 4.95 mm. A senior radiologist with 16 years of experience was invited to the delineation of the spinal metastasis (region of interest, ROI) border on each slice of the TIW and T2FS MRI image, using the ITK-SNAP software (version 3.6.0). All ROI segmentations were saved in an NII format.

### Radiomics feature extraction and selection

Before the feature extraction, standarded preprocessing of the MRI images were performed, which include normalization, resampling, discretization and filtering of the images. Detailed descriptions have been shown in Supplementary 1. We evaluated 1967 features for each MRI sequence using the pyradiomics package (25) in Python v.3.6. The computational features consist of original features and filtered features. The original features include first-order statistical, shape and texture features. The texture features contain gray level co-occurence matrix (GLCM), gray level run length matrix (GLRLM), gray level size zone matrix (GLSZM), neighbour gray tone difference matrix (NGTDM) and gray level dependence matrix (GLDM). To obtain high dimensional features, the original MR images were filtered with wavelet, square, exponential, squareroot, gradient, logarithm, localbinarypattern 2D/3D, and Laplacian of gaussian filters. Then, first-order features and texture features are obtained from these conversions. Details about the feature calculation protocols were provided in the Pyradiomics document (available from URL: https://pyradiomics.readthedocs.io/) and in a prior report (26) (27).

Another radiologist independently manually segmented the metastases in the MRI images of 30 randomly selected patients to evaluate the feature consistency expressed by the inter-class correlation coefficient (ICC) (28). High values (ICC > 0.8) indicate that results of independent evaluations by different observers are consistent. All features with *P*< 0.05 were selected using the Mann - Whitney *U* test. Then, the least absolute shrinkage and selection operator (LASSO) algorithm was employed to identify the most predictive features according to their associations with the EGFR mutation and subtypes with 5-fold cross-validation using the glmnet package in R language v3.6 (available from URL: https://www.r-project.org). The extracted features were further picked using the Max-Relevance and Min-Redundancy (mRMR) (29). Finally, the remained features were treated with the logistic regression (30) with Akaike Information Criterion (AIC) as the stopping rule.

### Construction and evaluation of the radiomics signature, clinical model and nomogram

**Figure 2:**
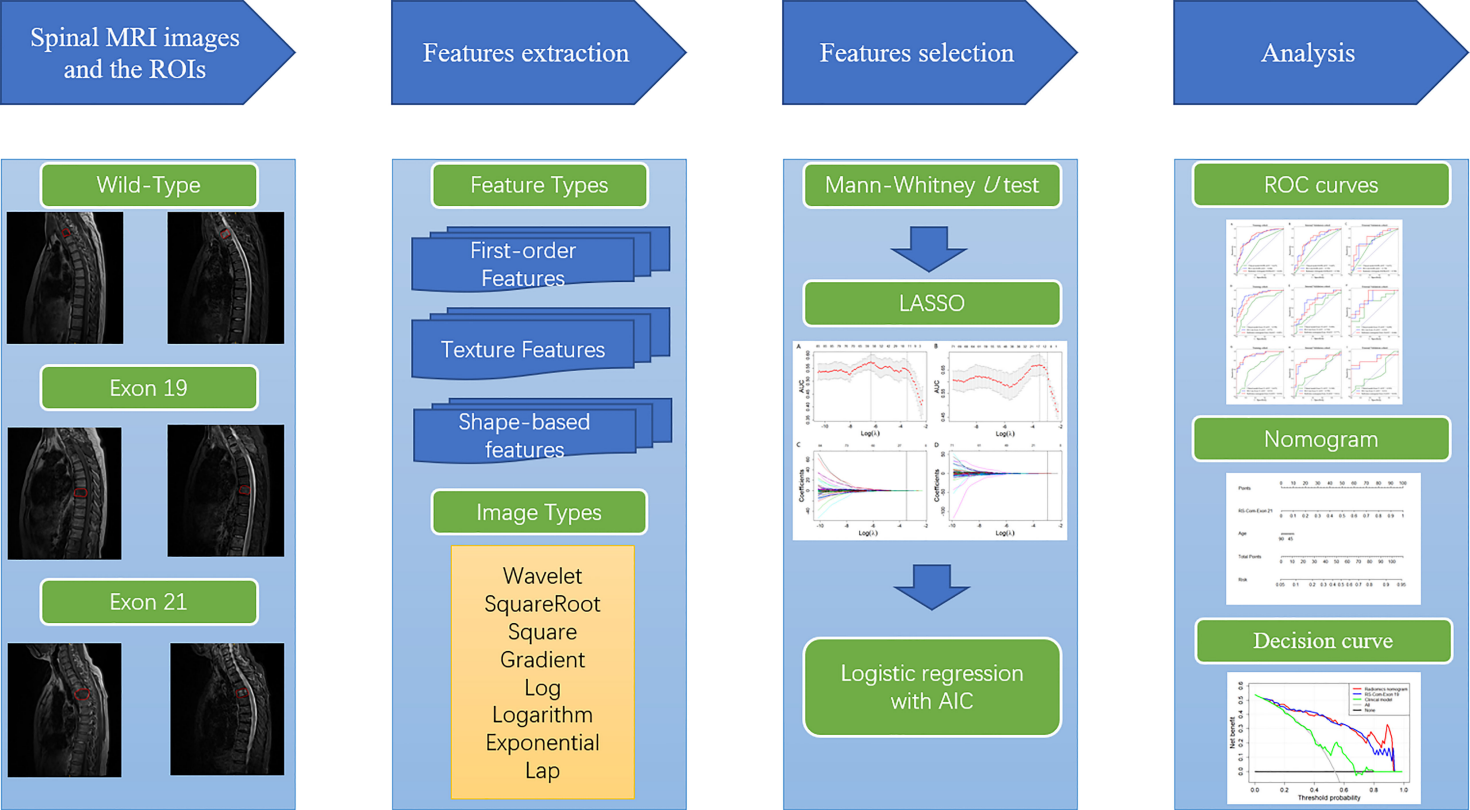
Overall workflow of this study.

Radiomics signatures (RSs) were established using the glmnet package in R v.3.6 with logistic regression. Clinical factors of P< 0.05 were determined by Mann - Whitney U and Chi - Square tests, and used to develop a clinical model. The clinical-radiomics nomogram was established by combing of the selected features and important clinical factors using the rms package in R. The models were evaluated using the receiver operating characteristic (ROC), calibration and decision curve analyses. The ROC curve was drawn using the proc package in R, with the best cut-off values evaluated by the Youden index (31). The calibration curve was drawn using the rms package to evaluate the consistency between the model-predicted and actual results. The clinical usefulness of the radiomics models was evaluated by quantifying the net benefits at different threshold probabilities using the rmda package to perform decision curve analysis (32). The overall workflow of this study was shown in **Figure 2**.

## Results

### Clinical characteristics

In the primary set, there were 60 patients carry EGFR mutation in exon 19, 65 patients carry EGFR mutation in exon 21, and 12 patients carry EGFR mutation in exon 18/20. The rest 120 patients in the primary cohort were with EGFR wild-type. In the external validation set, there were 18, 11, 11 and 2 patients carry the EGFR wild-type and EGFR mutations in exon 19, 21, and 18/20, respectively. According to the univariate analysis, smoking was the important influencing factor related to the EGFR mutation (*P*<0.05). The exon 19 mutation is related to smoking and age (*P*<0.05). While, the exon 21 mutation is related to age (*P*<0.05). **Table 1** presents the statistical analysis of the clinical characteristics. Results of univariate analysis for smoking and age were provided in Supplementary 2.

**Table 1:** Clinical characteristics of all patients in the training, internal validation and external validation sets.

Characteristic	Training (n = 171)			Internal validation (n = 86)			External validation (n = 42)		
	EGFR + (n = 91)	EGFR - (n = 80)	*P*	EGFR + (n = 46)	EGFR - (n = 40)	*P*	EGFR + (n = 24)	EGFR - (n = 18)	*P*
Age (Mean ± SD)	60.19 ± 9.99	59.28 ± 10.35	0.998	61.15 ± 11.77	59.60 ± 10.42	0.428	60.17 ± 7.63	60.06 ± 6.78	0.721
Gender, n (%)			0.661			0.841			0.107
Male	47 (51.4%)	44 (55.0%)		22 (47.8%)	20 (50.0%)		8 (33.3%)	9 (50.0%)	
Female	44 (48.6%)	36 (45.0%)		24 (52.2%)	20 (50.0%)		16 (66.7%)	9 (50.0%)	
Smoking, n (%)			<0.001*			0.039*			0.021*
Yes	22 (24.2%)	41 (51.3%)		11 (23.9%)	18 (45.0%)		6 (37.5%)	8 (44.4%)	
No	69 (75.8%)	39 (48.7%)		35 (76.1%)	22 (55.0%)		10 (62.5%)	10 (65.6%)	
PS score			0.718			0.144			0.310
0	7 (7.7%)	4 (5.0%)		7 (15.2%)	1 (2.5%)		6 (25.0%)	5 (27.8%)	
1	70 (76.9%)	65 (81.2%)		28 (60.9%)	32 (80.0%)		15 (62.5%)	11 (61.1%)	
2	11 (12.1%)	10 (12.5%)		9 (19.6%)	5 (12.5%)		3 (12.5%)	2 (11.1%)	
3	3 (3.3%)	1 (1.3%)		2 (4.3%)	2 (5.0%)		0 (0.00%)	0 (0.00%)	
CEA (Mean ± SD)	121.40 ± 177.29	88.92 ± 176.06	0.423	110.27 ± 177.98	88.90 ± 148.75	0.979	70.69 ± 129.72	16.11 ± 15.65	0.128
CYFRA (Mean ± SD)	12.04 ± 19.61	6.63 ± 6.46	0.02*	11.33 ± 19.76	7.03 ± 7.35	0.445	77.80 ± 143.02	5.757 ± 3.22	0.087
NSE (Mean ± SD)	20.13 ± 19.00	19.10 ± 13.68	0.867	18.10 ± 9.67	16.93 ± 7.24	0.404	55.35 ± 86.55	41.74 ± 80.19	0.381

PS score, performance status score; CEA, carcinoembryonic antigen; CYFRA, cytokeratin; NSE, neuron specific enolase; *, P<0.05.

### Feature selection and RS development

**Figure 3:**
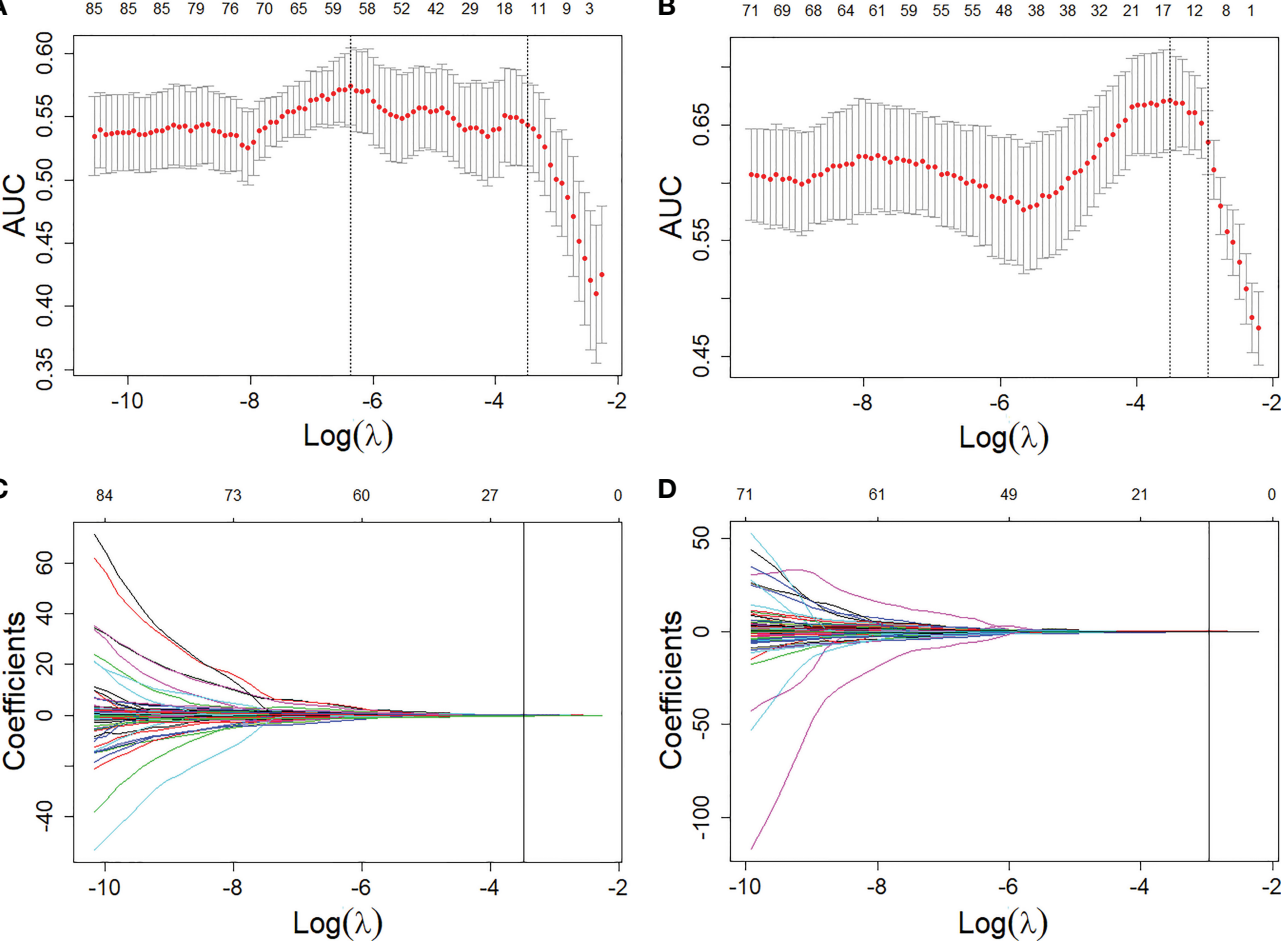
Feature selections from the T1W and T2FS MRI with LASSO. **(A, B)**, LASSO coefficient analyses of the features with 5-fold cross-validation for selecting optimal log ( λ ) in T1W **(A)** and T2FS **(B)** MRI, respectively. **(C, D)**, LASSO coefficients plotted against the log ( λ ) sequence in T1W **(C)** and T2FS **(D)** MRI, respectively.

A total of 24 features were selected as the most important predictors to detect the EGFR mutation, 12 from T1W and 12 from T2FS MRI, and used to build the RS-EGFR-T1W and RS-EGFR-T2FS, respectively. Similarly, fourteen features were selected from the T1W (7 features) and T2FS (7 features) to locate the exon 19 mutation, and used to established the RS-Exon 19-T1W and RS-Exon 19-T2FS, respectively. To detect the exon 21 mutation, fourteen features were selected from T1W (7 features) and T2FS (7 features), and used to build RS-Exon 21-T1W and RS-Exon 21-T2FS, respectively. **Figure 3** showed the selection of radiomics features using the LASSO regression. Prediction performance of each RS were compared and listed in **Table 2**. The Exons (Exon 19 and 21) were compared within the EGFR mutation group. RSs derived from T1W MRI generated higher values of AUC, accuracy and specificity compared with those from T2FS MRI, for detecting the EGFR mutation and subtypes.

**Table 2:** Comparisons of the developed RSs for detecting the EGFR mutation and subtypes.

		Training					Internal validation					External validation				
		AUC	Acc	Spe	Sen	*P*	AUC	Acc	Spe	Sen	*P*	AUC	Acc	Spe	Sen	*P*
**EGFR** **Mutation**	**RS-EGFR-T1W**	0.756	0.740	0.725	0.714		0.742	0.738	0.850	0.652		0.729	0.698	0.771	0.679	
	**RS-EGFR-T2FS**	0.753	0.716	0.688	0.754		0.682	0.696	0.650	0.696		0.697	0.672	0.697	0.724	
	**RS-Com-EGFR**	0.806	0.732	0.863	0.670		0.745	0.704	0.725	0.696		0.738	0.709	0.755	0.687	
	**RS-EGFR-T1W** **vs.** **RS-EGFR-T2FS**					0.381					0.109					0.279
**Exon 19**	**RS-Exon 19-T1W**	0.840	0.737	0.775	0.784		0.758	0.720	0.750	0.692		0.819	0.711	0.731	0.742	
	**RS-Exon 19-T2FS**	0.828	0.702	0.700	0.863		0.754	0.715	0.750	0.731		0.801	0.709	0.728	0.857	
	**RS-Com-Exon 19**	0.872	0.762	0.700	0.922		0.760	0.758	0.550	0.962		0.825	0.736	0.739	0.869	
	**RS-Exon 19-T1W** **vs.** **RS-Exon 19-T2FS**					0.447					0.376					0.397
**Exon 21**	**RS-Exon 21-T1W**	0.854	0.781	0.767	0.812		0.758	0.721	0.727	0.792		0.804	0.719	0.732	0.807	
	**RS-Exon 21-T2FS**	0.835	0.769	0.721	0.812		0.722	0.690	0.818	0.708		0.746	0.713	0.729	0.736	
	**RS-Com-Exon 21**	0.913	0.780	0.837	0.875		0.799	0.771	0.864	0.750		0.811	0.723	0.714	0.786	
	**RS-Exon 21-T1W** **vs.** **RS-Exon 21-T2FS**					0.501					0.318					0.401

The first column is the models detecting EGFR mutation, Exon 19 and Exon 21. The second column is the model established by T1W sequence, T2FS sequence, and combined of two sequences to predict EGFR mutation, Exon 19 and Exon 21.

### Development and validation of combined RSs

From two MRI sequences, 3934 (1967×2) were extracted and selected by ICC analysis, Mann-Whitney *U* test, mRMR and LASSO with AIC. A total of 8 features were selected and used to develop a RS-Com-EGFR for detecting the EGFR mutation. For detections of the exon 19 and exon 21 mutations, 9 and 8 features were selected and used to develop a RS-Com-Exon 19 and a Rs-Com-Exon 21 for detecting the exon 19 and exon 21 mutations, respectively. Supplementary 3 listed the finally retained features and their prediction performance to detecting the EGFR mutation and subtypes.

As shown in **Table 2**, the combined radiomics signatures always generated higher AUC, accuracy and specificity compared with those from single MRI sequence, which indicated that the multi-parametric MRI combined radiomics signature can effectively detect the EGFR mutation and subtypes.

Formulas of the RS-Com-EGFR, RS-Com Exon 19 and RS-Com Exon 21 were shown as following:


$$\begin{array}{l} \text{RS}-\text{Com}-\text{EGFR}=4.161-7.881\times \\ \text{wavelet}\_\text{LHL}\_\text{glszm}\_\text{SmallAreaLowGrayLevelEmphasis}\\ +3.981\times \\ \text{wavelet}\_\text{LLH}\_\text{firstorder}\_\text{Skewness}+4.868\times \\ \text{wavelet}\_\text{LHH}\_\text{glrlm}\_\text{ShortRunLowGrayLevelEmphasis}\\ +7.198\times \\ \text{log}\_\text{sigma}\_3\_0\_\text{mm}\_3\text{D}\_\text{firstorder}\\ \_\text{TotalEnergy}-5.626\times \text{gradient}\_\text{glcm}\_\\ \text{Correlation}-3.305\times \\ \text{log}\_\text{sigma}\_1\_0\_\text{mm}\_3\text{D}\\ \_\text{glszm}\_\text{SmallAreaLowGrayLevelEmphasis}+9.492\times \\ \text{wavelet}\_\text{HLH}\_\text{firstorder}\_\text{Mean}-3.082\times \\ \text{wavelet}\_\text{HHH}\_\text{glszm}\_\text{LowGrayLevelZoneEmphasis} \end{array}$$

$$\begin{array}{l} \text{RS}-\text{Com}-\text{Exon }19=83.9707-79.94451\times \\ \text{logarithm}\_\text{glszm}\_\text{LowGrayLevelZoneEmphasis}-237.98322\times \\ \text{wavelet}\_\text{HHH}\_\text{glcm}\_\text{InverseVariance}-7.69075\times \text{wavelet}\_\text{HHL}\\ \_\text{gldm}\_\text{SmallDependenceHighGrayLevelEmphasis}-17.05724\times \\ \text{lbp}\_3\text{D}\_\text{k}\_\text{glcm}\_\text{ClusterShade}+2.25838\times \text{lbp}\_3\text{D}-\text{m}2\_\\ \text{firstorder}\_90\text{Percentile}+0.74679\times \\ \text{log}\_\text{sigma}\_1\_0\_\text{mm}\_3\text{D}\_\text{firstorder}\_\text{Skewness}+6.17158\times \\ \text{logarithm}\_\text{glcm}\_\text{Imc}2-25.50497\times \\ \text{log}\_\text{sigma}\_5\_0\_\text{mm}\_3\text{D}\_\text{gldm}\_\\ \text{DependenceNonUniformityNormalized}+0.02124\times \\ \text{wavelet}\_\text{HLH}\_\text{firstorder}\_\text{Maximum} \end{array}$$

$$\begin{array}{l} \text{RS}-\text{Com}-\text{Exon }21=27.55874+5.62779\times \text{lbp}\_3\text{D}\_\text{k}\_\\ \text{firstorder}\_\text{Variance}-11.49092\times \\ \text{wavelet}\_\text{LHH}\_\text{glszm}\_\text{SmallAreaLowGrayLevelEmphasis}+\\ 0.01118\times \text{wavelet}-\text{LLL}\_\text{firstorder}\_\text{Minimum}-12.83371\times \\ \text{gradient}\_\text{glcm}\_\text{Imc}2+0.15562\times \text{wavelet}-\text{LLH}\_\text{firstorder}\\ \_\text{Median}-23.67674\times \text{wavelet}\_\text{HHL}\_\text{glcm}\_\text{Idmn}+56.93538\times \\ \text{lbp}\_3\text{D}\_\text{k}\_\text{glcm}\_\text{Imc}2-15.75006\times \text{wavelet}\_\text{HLH}\_\text{glcm}\_\text{MCC} \end{array}$$

### Development and valuation of the radiomics nomogram

**Figure 4:**
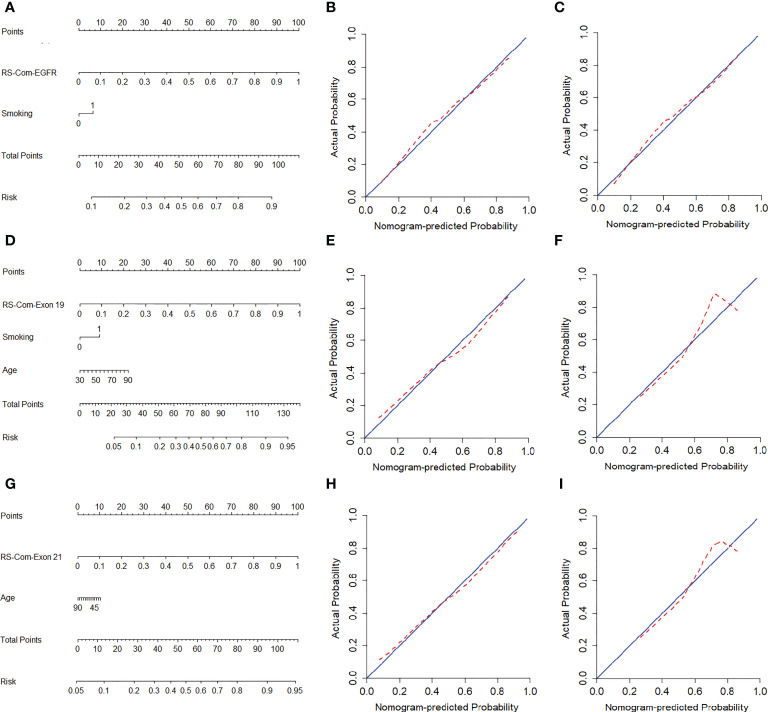
Nomogram models for predicting the EGFR mutation and subtypes. **(A–C)**, nomogram for predicting the EGFR mutation. calibration curves of nomogram in the training **(B)** and internal validation **(C)** set. **(D–F)**, nomogram for predicting the exon 19 mutation. calibration curves of nomogram in the training **(E)** and internal validation **(F)** set. **(G–I)**, nomogram for predicting the exon 21 mutation. Calibration curves of nomogram in the training **(H)** and internal validation **(I)** set. The red dotted line indicates the nomogram-predicted performance, whereas the blue line indicates an ideal prediction.

**Figure 5:**
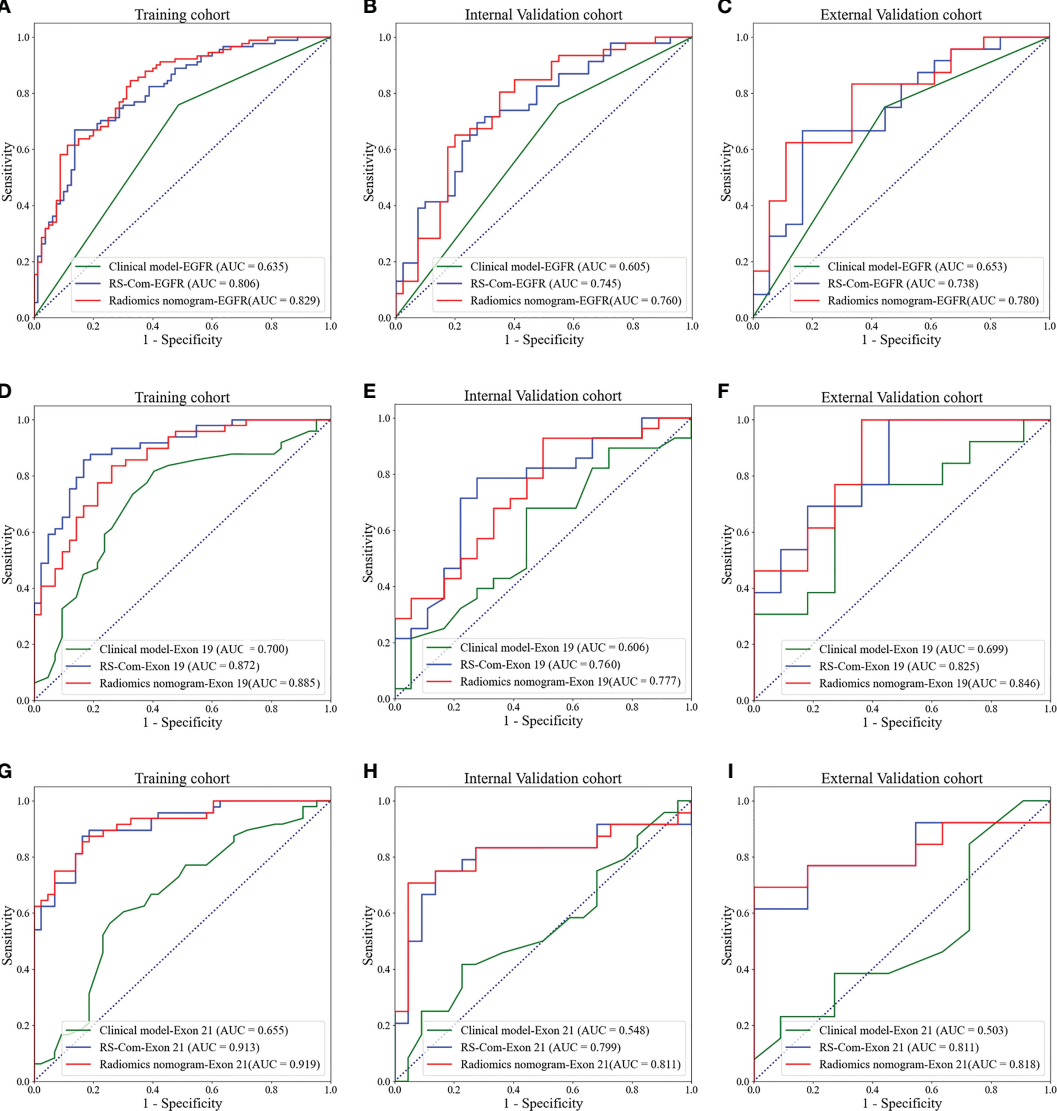
ROC curves for predicting the EGFR mutations and subtypes. **(A–C)**, ROC curves of the models for predicting the EGFR mutation in the training **(A)**, internal validation **(B)** and external validation **(C)** cohorts. **(D–F)**, ROC curves of the models for predicting the EGFR mutation in exon 19 in the training **(D)**, internal validation **(E)** and external validation **(F)** cohorts. **(G–I)**, ROC curves of the models for predicting the EGFR mutation in exon 21 in the training **(G)**, internal validation **(H)** and external validation **(I)** cohorts.

**Figure 6:**
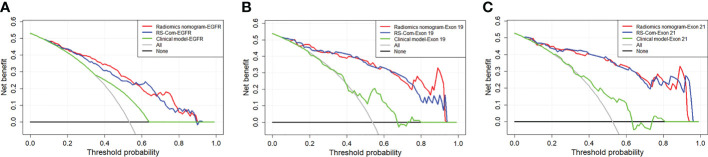
DCA curves for the developed radiomics models in the training **(A)**, internal validation **(B)** and external validation **(C)** cohorts.

**Figure 4A** showed the developed nomogram models incorporating the combined RS-Com-EGFR and smoking to detect the EGFR mutation. **Figure 4D** demonstrated the nomogram based on the RS-Com-Exon19, smoking and age to locate the exon 19 mutation site. **Figure 4G** exhibited the nomogram consists of RS-Com-Exon 21 and age to locate the exon 21 mutation site. Calibration curves (**Figures 4B, C, E, F, H, I**) proved whether the predicted values of the nomogram models were consistent with actual values. **Figure 5** shows ROC curves of the developed models. For predicting the EGFR mutation, the Clinical model-EGFR, Rs-Com-EGFR and Radiomics nomogram-EGFR were developed by using smoking status, combination of T1W and T2FS MRI, and integration of smoking status with RS-Com-EGFR, respectively. For predicting the Exon 19 mutation, the Clinical model-Exon 19, Rs-Com-Exon 19 and Radiomics nomogram-Exon 19 were developed by using smoking and age, combination of T1W and T2FS MRI, and integration of smoking and age with Rs-Com-Exon-19, respectively. For predicting the Exon 21 mutation, the Clinical model-Exon 21, Rs-Com-Exon 21 and Radiomics nomogram-Exon 21 were developed by using age, combination of T1W and T2FS MRI, and integration of age with Rs-Com-Exon-21, respectively. The nomogram models always outperformed the combined radiomics signatures and clinical models for predicting the EGFR mutation and subtypes. **Table 3** compares prediction capabilities of each model. For predicting the EGFR mutations in exon 21, the clinical models were significantly different (*P*<0.05) from the nomogram models by the Delong test. **Figure 6** depicts the decision curves of each model, which shows that our nomogram can obtain better prediction performance on judging whether the patient is carrying the EGFR mutation and the mutation subtypes.

**Table 3:** Comparisons of the combined radiomics signatures, clinical models and nomogram models.

	Training					Internal validation					External validation				
	AUC	Acc	Spe	Sen	*p*	AUC	Acc	Spe	Sen	*p*	AUC	Acc	Spe	Sen	*p*
M1	0.806	0.732	0.863	0.670		0.745	0.704	0.725	0.696		0.738	0.709	0.755	0.687	
M2	0.635	0.601	0.758	0.512		0.605	0.616	0.761	0.550		0.653	0.637	0.720	0.614	
M3	0.829	0.747	0.675	0.846		0.760	0.792	0.650	0.804		0.780	0.728	0.754	0.673	
M1 vs M2					0.040					0.265					0.106
M1 vs M3					0.384					0.637					0.249
M2 vs M3					0.009					0.157					0.103
M4	0.872	0.762	0.700	0.922		0.760	0.758	0.550	0.962		0.825	0.736	0.739	0.869	
M5	0.700	0.618	0.600	0.784		0.606	0.587	0.600	0.692		0.699	0.653	0.651	0.766	
M6	0.885	0.813	0.900	0.725		0.777	0.791	0.650	0.808		0.846	0.801	0.744	0.795	
M4 vs. M5					0.162					0.247					0.199
M4 vs. M6					0.379					0.674					0.324
M5 vs. M6					0.076					0.045					0.017
M7	0.913	0.780	0.837	0.875		0.799	0.771	0.864	0.750		0.811	0.723	0.714	0.786	
M8	0.655	0.608	0.744	0.562		0.548	0.597	0.773	0.583		0.503	0.559	0.608	0.697	
M9	0.919	0.830	0.837	0.854		0.811	0.782	0.955	0.708		0.818	0.801	0.846	0.800	
M7 vs M8					0.032					0.176					0.018
M7 vs M9					0.563					0.612					0.753
M8 vs M9					0.011					0.027					0.044

M1, RS-Com-EGFR; M2, Clinical model-EGFR; M3, Nomogram-EGFR; M4, RS-Com-Exon 19; M5, Clinical model-Exon 19; M6, Nomogram-Exon19; M7, RS-Com-Exon 21; M8, Clinical model-Exon 21; M9, Nomogram-Exon 21.

## Discussion

In this study, values of multi-parametric MRI-based radiomics for assessment of the EGFR mutation and subtypes based on the spinal metastasis were analyzed. We found that the developed RSs derived from T1W generated higher AUC, accuracy and specificity compared with those from T2FS for predicting the EGFR mutation and subtypes. This may be explainable since the T1W MRI can reflect the anatomical structure, which is helpful to show the deep-seated information within the tumor area. While, the T2FS MRI reflects high signal intensities of the metastasis by suppressing fat hyperintensities of the bone marrow. The combined RSs based on the combination of the two MRI sequences can improve the predictive performance in regard to AUC values, which may be because complementary information can be obtained from the two modalities. There were recent studies related to our work, Jiang et al. and Ren et al. previously proposed machine learning models to predict the EGFR mutation based on the bone metastasis in lumbar (33) and thoracic spine (34), respectively. While, the reports only explored radiomics approaches to evaluate the overall mutation status of the *EGFR* gene, and failed to predict the mutation subtypes. Besides, the studies were lack of external validation sets, which inhered their clinical values. A previous study evaluated values of radiomics for differentiating EGFR mutations in exon 19 and exon 21 (24). However, the study has inherent bias with a limited sample size (n=76) from a single center. Different from the previous study, this work enrolled 299 patients from two centers and comprehensively explored multiparameter MRI-based radiomics for predicting the EGFR mutations (exon 18/19/20/21) and exon 19/21.

We finally selected a total of 8, 9 and 8 most predictive features for predicting the EGFR mutation, exon 19 and exon 21, respectively. All features belong to the first-order and textural feature categories. The first-order feature quantifies the distribution of voxel intensity (35). While, the textural feature (36) calculates the thickness of the tumor texture, which reflects the heterogeneity within the tumor. The majority of the features (18 of 25) were textural features, which may suggest that the intertumoral heterogeneity might be highly related to the EGFR mutation and subtypes. All predictive features belong to the filtered features that cannot be detected by radiologists (37). This may explain why radiologists can hardly evaluate the EGFR mutation status by visual observations on MRI data of the bone metastasis.

Some clinical factors, such as gender and smoking, have been shown to be associated with the EGFR mutation in NSCLC (38) (39). Some studies showed that the smoking and EGFR mutation subtypes are highly correlated (38) (40). In this study, we found that the smoking is highly correlated with the EGFR mutation. For locating the EGFR mutation sites, the smoking and age were found to be independent predictive factors. While, the age was previously considered invalid for predicting the EGFR subtypes (39). To explore potential values of the clinical factors, we integrated important clinical factors with combined radiomics signatures to construct nomogram models (41), which significantly improved the prediction performance. This suggests that important clinical factors and imaging features may be complement. Comparisons of each model by DCA further proves that our nomogram has better clinical applicability (42). Therefore, we believe that our nomogram can be used as an effective non-invasive tool to detect the EGFR mutation and subtypes in NSCLC patients with bone metastasis.

This study has some limitations. First, although we included an independent validation set from another center, the amount of samples was still small and the ethnic group was single. Second, Some serum biomarkers were not included in this study due to missing clinical data. Finally, this study only assessed the EGFR mutation and subtypes. Other important mutation types (e.g. KRAS, ALK and ROS1) that were also important for the treatment planning in NSCLC were not assessed due to data collection challenges.

## Conclusion

In conclusion, we developed and externally validated multi-parametric MRI-based radiomics to predict the EGFR mutation and subtypes. The constructed nomogram provide a potential non-invasive method that may help clinicians to make individualized treatment for NSCLC patients.

## Data availability statement

The original contributions presented in the study are included in the article/**Supplementary Materials**. Further inquiries can be directed to the corresponding authors.

## Ethics statement

The studies involving human participants were reviewed and approved by the Cancer Hospital of China Medical University. Written informed consent for participation was not required for this study in accordance with the national legislation and the institutional requirements.

## Author contributions

RC and WJ: study design. RC, HC and HW: data collection. RC, YW and E-NC: data analysis and interpretation. RC and WJ: manuscript writing. WJ and E-NC: funding acquisition. All authors contributed to the article and approved the submitted version.

## References

[1] Bray F Ferlay J Soerjomataram I Siegel RL Torre LA Jemal A. Global cancer statistics 2018: GLOBOCAN estimates of incidence and mortality worldwide for 36 cancers in 185 countries. CA Cancer J Clin (2018) 68(6):394–424. doi: 10.3322/caac.21492 30207593

[2] Ferlay J Soerjomataram I Dikshit R Eser S Mathers C Rebelo M. Cancer incidence and mortality worldwide: Sources, methods and major patterns in GLOBOCAN 2012. Int J Cancer. (2015) 136(5):E359–86. doi: 10.1002/ijc.29210 25220842

[3] Cheng T Zhang Z Cheng Y Zhang J Tang J Tan Z. ETV4 promotes proliferation and invasion of lung adenocarcinoma by transcriptionally upregulating MSI2. Biochem Biophys Res Commun (2019) 516(1):278–84. doi: 10.1016/j.bbrc.2019.06.115 31253395

[4] Yang JC Wu YL Schuler M Sebastian M Popat S Yamamoto N. Afatinib versus cisplatin-based chemotherapy for EGFR mutation-positive lung adenocarcinoma (LUX-lung 3 and LUX-lung 6): analysis of overall survival data from two randomised, phase 3 trials. Lancet Oncol (2015) 16:141–51. doi: 10.1016/S1470-2045(14)71173-8 25589191

[5] Liang SK Ko JC Yang JC Shih JY. Afatinib is effective in the treatment of lung adenocarcinoma with uncommon EGFR p.L747P and p.L747S mutations. Lung Cancer (2019) 133:103–9. doi: 10.1016/j.lungcan.2019.05.019 31200815

[6] Huang YH Hsu KH Chin CS Tseng JS Yang TY Chen KC. The clinical outcomes of different first-line EGFR-TKIs plus bevacizumab in advanced EGFR-mutant lung adenocarcinoma. Cancer Res Treat (2022) 54(2):434–44. doi: 10.4143/crt.2021.671 PMC9016311 34352999

[7] Li S Luo T Ding C Huang Q Guan Z Zhang H. Detailed identification of epidermal growth factor receptor mutations in lung adenocarcinoma: Combining radiomics with machine learning. Med Phys (2020) 47(8):3458–66. doi: 10.1002/mp.14238 32416013

[8] Li S Ding C Zhang H Song J Wu L. Radiomics for the prediction of EGFR mutation subtypes in non-small cell lung cancer. Med Phys (2019) 46(10):4545–52. doi: 10.1002/mp.13747 31376283

[9] Yano M Sasaki H Kobayashi Y Yukiue H Haneda H Suzuki E. Epidermal growth factor receptor gene mutation and computed tomographic findings in peripheral pulmonary adenocarcinoma. J Thorac Oncol (2006) 1:413–6. doi: 10.1097/01243894-200606000-00006 17409892

[10] Carey KD Garton AJ Romero MS Kahler J Thomson S Ross S. Kinetic analysis of epidermal growth factor receptor somatic mutant proteins shows increased sensitivity to the epidermal growth factor receptor tyrosine kinase inhibitor, erlotinib. Cancer Res (2006) 66(16):8163–71. doi: 10.1158/0008-5472.CAN-06-0453 16912195

[11] Krawczyk P Nicoś M Ramlau R Powrózek T Wojas-Krawczyk K Sura S. The incidence of EGFR-activating mutations in bone metastases of lung adenocarcinoma. Pathol Oncol Res (2014) 20(1):107–12. doi: 10.1007/s12253-013-9667-4 PMC3889871 23852459

[12] Shen TX Liu L Li WH Fu P Xu K Jiang YQ. CT imaging-based histogram features for prediction of EGFR mutation status of bone metastases in patients with primary lung adenocarcinoma. Cancer Imaging. (2019) 19(1):34. doi: 10.1186/s40644-019-0221-9 31174617 PMC6556025

[13] Song J Shi J Dong D Fang M Zhong W Wang K. A new approach to predict progression-free survival in stage IV EGFR-mutant NSCLC patients with EGFR-TKI therapy. Clin Cancer Res (2018) 24(15):3583–92. doi: 10.1158/1078-0432.CCR-17-2507 29563137

[14] Han X Fan J Gu J Li Y Yang M Liu T. CT features associated with EGFR mutations and ALK positivity in patients with multiple primary lung adenocarcinomas. Cancer Imaging. (2020) 20(1):51. doi: 10.1186/s40644-020-00330-1 32690092 PMC7372851

[15] Song J Wang L Ng NN Zhao M Shi J Wu N. Development and validation of a machine learning model to explore tyrosine kinase inhibitor response in patients with stage IV EGFR variant-positive non-small cell lung cancer. JAMA Netw Open (2020) 3(12):e2030442. doi: 10.1001/jamanetworkopen.2020.30442 33331920 PMC7747022

[16] Cao R Pang Z Wang X Du Z Chen H Liu J. Radiomics evaluates the EGFR mutation status from the brain metastasis: a multi-center study. Phys Med Biol (2022) 67(12). doi: 10.1088/1361-6560/ac7192 35588722

[17] Fan Y Dong Y Wang H Wang H Sun X Wang X. Development and externally validate MRI-based nomogram to assess EGFR and T790M mutations in patients with metastatic lung adenocarcinoma. Eur Radiol (2022) 32(10):6739–6751. doi: 10.1007/s00330-022-08955-5 35729427

[18] Digumarthy SR Padole AM Gullo RL Sequist LV Kalra MK. Can CT radiomic analysis in NSCLC predict histology and EGFR mutation status? Medicine (2019) 98:1–9. doi: 10.1097/MD.0000000000013963 PMC6344142 30608433

[19] Zhang L Chen B Liu X Song J Fang M Hu C. Quantitative biomarkers for prediction of epidermal growth factor receptor mutation in non-small cell lung cancer. Transl Oncol (2018)():– 11:94–101. doi: 10.1016/j.tranon.2017.10.012 29216508 PMC6002350

[20] Mu W Jiang L Zhang J Shi Y Gray JE Tunali I. Non-invasive decision support for NSCLC treatment using PET/CT radiomics. Nat Commun (2020) 11(1):5228. doi: 10.1038/s41467-020-19116-x 33067442 PMC7567795

[21] Wang G Wang B Wang Z Li W Xiu J Liu Z. Radiomics signature of brain metastasis: prediction of EGFR mutation status. Eur Radiol (2021) 31(7):4538–47. doi: 10.1007/s00330-020-07614-x 33439315

[22] Ahn SJ Kwon H Yang JJ Park M Cha YJ Suh SH. Contrast-enhanced T1-weighted image radiomics of brain metastases may predict EGFR mutation status in primary lung cancer. Sci Rep (2020) 10(1):8905. doi: 10.1038/s41598-020-65470-7 32483122 PMC7264319

[23] Wang S Shi J Ye Z Dong D Yu D Zhou M. Predicting EGFR mutation status in lung adenocarcinoma on computed tomography image using deep learning. Eur Respir J (2019) 53(3):1800986. doi: 10.1183/13993003.00986-2018 30635290 PMC6437603

[24] Cao R Dong Y Wang X Ren M Wang X Zhao N. MRI-Based radiomics nomogram as a potential biomarker to predict the EGFR mutations in exon 19 and 21 based on thoracic spinal metastases in lung adenocarcinoma. Acad Radiol (2021) 29(3):e9–e17. doi: 10.1016/j.acra.2021.06.004 34332860

[25] Lee CI Lehman CD. Digital breast tomosynthesis and the challenges of implementing an emerging breast cancer screening technology into clinical practice. J Am Coll Radiol (2016) 13(11S):R61–6. doi: 10.1016/j.jacr.2016.09.029 27814817

[26] Fan Y Dong Y Yang H Chen H Yu Y Wang X. Subregional radiomics analysis for the detection of the EGFR mutation on thoracic spinal metastases from lung cancer. Phys Med Biol (2021) 66(21). doi: 10.1088/1361-6560/ac2ea7 34633298

[27] van Griethuysen JJM Fedorov A Parmar C Hosny A Aucoin N Narayan V. Computational radiomics system to decode the radiographic phenotype. Cancer Res (2017) 77(21):e104–e107. doi: 10.1158/0008-5472.CAN-17-0339 29092951 PMC5672828

[28] Li Z Duan H Zhao K Ding Y. Stability of MRI radiomics features of hippocampus: An integrated analysis of test-retest and inter-observer variability. IEEE Access (2019) 7:97106–16. doi: 10.1109/ACCESS.2019.2923755

[29] Chen M He X Duan S Deng Y. A novel gene selection method based on sparse representation and max-relevance and Min-redundancy. Comb Chem High Throughput Screen (2017) 20(2):158–63. doi: 10.2174/1386207320666170126114051 28128052

[30] Wang QQ Yu SC Qi X Hu YH Zheng WJ Shi JX. Overview of logistic regression model analysis and application. Zhonghua Yu Fang Yi Xue Za Zhi (2019) 53(9):955–60. doi: 10.3760/cma.j.issn.0253-9624.2019.09.018 31474082

[31] Njor SH Andersen B Friis-Hansen L de Haas N Linnemann D Nørgaard H. The optimal cut-off value in fit-based colorectal cancer screening: An observational study. Cancer Med (2021) 10(5):1872–9. doi: 10.1002/cam4.3761 PMC7940214 33534955

[32] Vickers AJ Holland F. Decision curve analysis to evaluate the clinical benefit of prediction models. Spine J (2021) 21(10):1643–8. doi: 10.1016/j.spinee.2021.02.024 PMC8413398 33676020

[33] Jiang X Ren M Shuang X Yang H Shi D Lai Q. Multiparametric MRI-based radiomics approaches for preoperative prediction of EGFR mutation status in spinal bone metastases in patients with lung adenocarcinoma. J Magn Reson Imaging (2021) 54(2):497–507. doi: 10.1002/jmri.27579 33638577

[34] Ren M Yang H Lai Q Shi D Liu G Shuang X. MRI-Based radiomics analysis for predicting the EGFR mutation based on thoracic spinal metastases in lung adenocarcinoma patients. Med Phys (2021) 48(9):5142–51. doi: 10.1002/mp.15137 34318502

[35] Peng W Liu C Xia S Shao D Chen Y Liu R. Thyroid nodule recognition in computed tomography using first order statistics. BioMed Eng Online (2017) 16(1):67. doi: 10.1186/s12938-017-0367-2 28592331 PMC5461692

[36] Di Cataldo S Ficarra E. Mining textural knowledge in biological images: Applications, methods and trends. Comput Struct Biotechnol J (2016) 15:56–67. doi: 10.1016/j.csbj.2016.11.002 27994798 PMC5155047

[37] Georgeson MA. From filters to features: location, orientation, contrast and blur. Ciba Found Symp (1994) 184:147–271. doi: 10.1002/9780470514610.ch8 7882752

[38] Boeckx B Shahi RB Smeets D De Brakeleer S Decoster L Van Brussel T. The genomic landscape of nonsmall cell lung carcinoma in never smokers. Int J Cancer. (2020) 146(11):3207–18. doi: 10.1002/ijc.32797 31745979

[39] Liu G Xu Z Ge Y Jiang B Groen H Vliegenthart R. 3D radiomics predicts EGFR mutation, exon-19 deletion and exon-21 L858R mutation in lung adenocarcinoma. Transl Lung Cancer Res (2020) 9(4):1212–24. doi: 10.21037/tlcr-20-122 PMC7481623 32953499

[40] Abdel-Rahman O. Smoking and EGFR status may predict outcomes of advanced NSCLC treated with PD-(L)1 inhibitors beyond first line: A meta-analysis. Clin Respir J (2018) 12(5):1809–19. doi: 10.1111/crj.12742 29115057

[41] Zhang G Zhang J Cao Y Zhao Z Li S Deng L. Nomogram based on preoperative CT imaging predicts the EGFR mutation status in lung adenocarcinoma. Transl Oncol (2021) 14(1):100954. doi: 10.1016/j.tranon.2020.100954 33232920 PMC7691609

[42] Vickers AJ van Calster B Steyerberg EW. A simple, step-by-step guide to interpreting decision curve analysis. Diagn Progn Res (2019) 3:18. doi: 10.1186/s41512-019-0064-7 31592444 PMC6777022

