# Supplementary material for: Comprehensive analysis of prediction of the EGFR mutation and subtypes based on the spinal metastasis from primary lung adenocarcinoma
Source: Front Oncol. 2023 Apr 18;13:1154327. doi: 10.3389/fonc.2023.1154327 (PMC10151709; doi:10.3389/fonc.2023.1154327)
Supplement: Supplementary file 1 [file Table_1.docx]

Table S1. Preprocessing steps before feature extraction.

| **Step** | **Description** |
| --- | --- |
| 1. Normalization | The normalization was performed based on gray level values in each MRI image. In detail, normalized the MRI image by centering it at the mean with standard deviation. The parameter “normalize” was set to be true to normalize the image before resampling. The parameter “normalizescale” was set to 100 to determine the scale after normalizing the image.  The normalizations of the gray level values in the MRI image were performed using the following function: $f(x)=\frac{s(x-\mu_{x})}{\sigma_{x}}$  where:  and $f(x)$ are the original and normalized intensity, respectively.  $\mu_{x}$ and $\sigma_{x}$ are the mean and standard deviation of the image intensity values.  The $s$ is an optional scaling defined by scale and was set to 1 by default. |
| 2. Resampling | The parameter “resampledpixelspacing” was set to [1,1,1] to determine the size of the voxel when resampling and use cubic B-spline interpolation to resample MRI images. |
| 3. Discretization | The parameter “binWith” was also set to 10 when making a histogram and discretizing the gray level of the images. |
| 4. Filtering | The original MRI images were transformed with eight types of filters to extract high-dimensional features, which include wavelet, laplacian of gaussian, square, squareroot, logarithm, exponential, gradient and local binary pattern 2D/3D filters. |
