# Supplementary material for: Comprehensive analysis of prediction of the EGFR mutation and subtypes based on the spinal metastasis from primary lung adenocarcinoma
Source: Front Oncol. 2023 Apr 18;13:1154327. doi: 10.3389/fonc.2023.1154327 (PMC10151709; doi:10.3389/fonc.2023.1154327)
Supplement: Supplementary file 2 [file Table_2.docx]

Table S2. Results of univariate analysis for smoking and age in primary and external cohorts.

| Task | Factor | Training  (*P*) | Internal validation  (*P*) | External validation  (*P*) |
| --- | --- | --- | --- | --- |
| EGFR mutation | Smoking | ＜0.001 | 0.039 | 0.021 |
| Exon 19 | Smoking | 0.007 | 0.028 | 0.019 |
|  | Age | ＜0.001 | 0.016 | 0.024 |
| Exon 21 | Age | 0.004 | 0.029 | 0.034 |
