# Supplementary material for: Comprehensive analysis of prediction of the EGFR mutation and subtypes based on the spinal metastasis from primary lung adenocarcinoma
Source: Front Oncol. 2023 Apr 18;13:1154327. doi: 10.3389/fonc.2023.1154327 (PMC10151709; doi:10.3389/fonc.2023.1154327)
Supplement: Supplementary file 3 [file Table_3.docx]

Table S3. The finally retained features and their prediction performance to detecting the EGFR mutation.

| **Features** | **Sources** | **Cohorts** | **Mean ± SD** | | **AUC** | ***P*** | **ICC** |
| --- | --- | --- | --- | --- | --- | --- | --- |
|  | | | **Mutation** | **Wild** |  | |  |
| wavelet-LHL_glszm_SmallAreaLowGrayLevelEmphasis | T1 | Training | 0.111±0.081 | 0.138±0.096 | 0.583 | 0.057 | 0.921 |
|  |  | Internal Validation | 0.111±0.094 | 0.131±0.093 | 0.586 | 0.337 |  |
|  |  | External Validation | 0.080±0.052 | 0.089±0.058 | 0.435 | 0.636 |  |
| wavelet-LLH_firstorder_Skewness | T1 | Training | -0.350±0.760 | -0.525±0.681 | 0.587 | 0.118 | 0.897 |
|  |  | Internal Validation | -0.266±0.627 | -0.504±0.772 | 0.596 | 0.127 |  |
|  |  | External Validation | -0.611±0.690 | -0.488±0.715 | 0.560 | 0.587 |  |
| wavelet-LHH_glrlm_ShortRunLowGrayLevelEmphasis | T1 | Training | 0.250±0.164 | 0.203±0.154 | 0.574 | 0.058 | 0.946 |
|  |  | Internal Validation | 0.261±0.158 | 0.212±0.161 | 0.558 | 0.171 |  |
|  |  | External Validation | 0.154±0.120 | 0.188±0.147 | 0.528 | 0.444 |  |
| log-sigma-3-0-mm-3D_firstorder_TotalEnergy | T1 | Training | 9.056$\times$10^6^±1.115$\times$10^7^ | 5.295$\times$10^6^±5.118$\times$10^6^ | 0.591 | 0.004 | 0.978 |
|  |  | Internal Validation | 1.797$\times$10^7^±4.266$\times$10^7^ | 5.908$\times$10^6^±5.673$\times$10^6^ | 0.611 | 0.066 |  |
|  |  | External Validation | 1.387$\times$10^7^±2.351$\times$10^7^ | 7.278$\times$10^6^±6.325$\times$10^6^ | 0.549 | 0.210 |  |
| gradient_glcm_Correlation | T2FS | Training | 0.373±0.096 | 0.406±0.117 | 0.582 | 0.052 | 0.874 |
|  |  | Internal Validation | 0.376±0.092 | 0.416±0.092 | 0.611 | 0.047 |  |
|  |  | External Validation | 0.344±0.112 | 0.294±0.065 | 0.678 | 0.085 |  |
| log-sigma-1-0-mm-3D_glszm_SmallAreaLowGrayLevelEmphasis | T2FS | Training | 0.012±0.008 | 0.018±0.022 | 0.574 | 0.014 | 0.944 |
|  |  | Internal Validation | 0.013±0.012 | 0.014±0.009 | 0.597 | 0.635 |  |
|  |  | External Validation | 0.014±0.014 | 0.014±0.011 | 0.523 | 0.989 |  |
| wavelet-HLH_firstorder_Mean | T2FS | Training | -0.058±0.303 | -0.251±0.645 | 0.574 | 0.016 | 0.897 |
|  |  | Internal Validation | -0.008±0.363 | -0.227±0.604 | 0.461 | 0.053 |  |
|  |  | External Validation | -0.271±0.550 | 0.019±0.222 | 0.639 | 0.028 |  |
| wavelet-HHH_glszm_LowGrayLevelZoneEmphasis | T2FS | Training | 0.512±0.178 | 0.587±0.155 | 0.637 | 0.004 | 0.916 |
|  |  | Internal Validation | 0.567±0.155 | 0.575±0.139 | 0.509 | 0.813 |  |
|  |  | External Validation | 0.340±0.166 | 0.394±0.212 | 0.442 | 0.389 |  |

Table S4. The finally retained features and their prediction performance to detecting the exon 19 mutation.

| **Features** | **Sources** | **Cohorts** | **Mean ± SD** | | **AUC** | ***P*** | **ICC** |
| --- | --- | --- | --- | --- | --- | --- | --- |
|  | | | **Exon 19** | **0thers** |  | |  |
| logarithm_glszm_LowGrayLevelZoneEmphasis | T1 | Training | 0.013±0.011 | 0.010±0.005 | 0.602 | 0.048 | 0.931 |
|  |  | Internal Validation | 0.016±0.015 | 0.015±0.014 | 0.506 | 0.866 |  |
|  |  | External Validation | 0.013±0.004 | 0.015±0.014 | 0.643 | 0.591 |  |
| wavelet-HHH_glcm_InverseVariance | T1 | Training | 0.513±0.002 | 0.511±0.003 | 0.651 | 0.020 | 0.910 |
|  |  | Internal Validation | 0.514±0.004 | 0.511±0.004 | 0.656 | 0.068 |  |
|  |  | External Validation | 0.508±0.021 | 0.505±0.026 | 0.454 | 0.760 |  |
| wavelet-HHL_gldm_SmallDependenceHighGrayLevelEmphasis | T1 | Training | 0.102±0.102 | 0.060±0.039 | 0.629 | 0.019 | 0.896 |
|  |  | Internal Validation | 0.081±0.058 | 0.057±0.029 | 0.610 | 0.112 |  |
|  |  | External Validation | 0.607±0.029 | 0.071±0.040 | 0.615 | 0.294 |  |
| lbp-3D-k_glcm_ClusterShade | T1 | Training | 0.190±0.044 | 0.160±0.044 | 0.692 | 0.003 | 0.924 |
|  |  | Internal Validation | 0.171±0.067 | 0.165±0.050 | 0.550 | 0.748 |  |
|  |  | External Validation | 0.177±0.066 | 0.209±0.057 | 0.615 | 0.250 |  |
| lbp-3D-m2_firstorder_90Percentile | T2FS | Training | 16.953±0.361 | 17.120±0.303 | 0.630 | 0.023 | 0.899 |
|  |  | Internal Validation | 19.093±0.385 | 17.180±0.243 | 0.488 | 0.396 |  |
|  |  | External Validation | 16.695±0.536 | 16.766±0.349 | 0.497 | 0.724 |  |
| log-sigma-1-0-mm-3D_firstorder_Skewness | T2FS | Training | -0.326±0.527 | -0.122±0.560 | 0.605 | 0.082 | 0.946 |
|  |  | Internal Validation | -0.445±0.445 | -0.301±0.453 | 0.596 | 0.298 |  |
|  |  | External Validation | -0.309±0.509 | -0.356±0.558 | 0.566 | 0.841 |  |
| logarithm_glcm_Imc2 | T2FS | Training | 0.720±0.107 | 0.754±0.082 | 0.609 | 0.099 | 0.971 |
|  |  | Internal Validation | 0.782±0.097 | 0.751±0.085 | 0.567 | 0.277 |  |
|  |  | External Validation | 0.814±0.098 | 0.781±0.113 | 0.615 | 0.470 |  |
| log-sigma-5-0-mm-3D_gldm_DependenceNonUniformityNormalized | T2FS | Training | 0.096±0.034 | 0.084±0.019 | 0.620 | 0.045 | 0.887 |
|  |  | Internal Validation | 0.125±0.090 | 0.088±0.022 | 0.583 | 0.101 |  |
|  |  | External Validation | 0.101±0.049 | 0.126±0.080 | 0.622 | 0.374 |  |
| wavelet-HLH_firstorder_Maximum | T2FS | Training | 89.466±30.643 | 109.930±44.868 | 0.640 | 0.012 | 0.961 |
|  |  | Internal Validation | 72.693±32.269 | 97.600±38.522 | 0.685 | 0.024 |  |
|  |  | External Validation | 277.175±132.294 | 282.196±154.602 | 0.510 | 0.935 |  |

Table S5. The finally retained features and their prediction performance to detecting the exon 21 mutation.

| **Features** | **Sources** | **Cohorts** | **Mean ± SD** | | **AUC** | ***P*** | **ICC** |
| --- | --- | --- | --- | --- | --- | --- | --- |
|  | | | **Exon 21** | **0thers** |  | |  |
| lbp-3D-k_firstorder_Variance | T1 | Training | 0.322±0.139 | 0.380±0.152 | 0.643 | 0.064 | 0.972 |
|  |  | Internal Validation | 0.326±0.121 | 0.357±0.145 | 0.466 | 0.444 |  |
|  |  | External Validation | 0.341±0.081 | 0.344±0.098 | 0.552 | 0.928 |  |
| wavelet-LHH_glszm_SmallAreaLowGrayLevelEmphasis | T1 | Training | 0.185±0.080 | 0.143±0.085 | 0.647 | 0.018 | 0.875 |
|  |  | Internal Validation | 0.180±0.088 | 0.108±0.097 | 0.735 | 0.013 |  |
|  |  | External Validation | 0.102±0.071 | 0.090±0.055 | 0.483 | 0.656 |  |
| wavelet-LLL_firstorder_Minimum | T1 | Training | -157.204±50.792 | -126.241±72.440 | 0.629 | 0.021 | 0.962 |
|  |  | Internal Validation | -155.001±62.800 | -121.346±82.177 | 0.587 | 0.132 |  |
|  |  | External Validation | -163.068±33.668 | -162.890±55.495 | 0.559 | 0.993 |  |
| gradient_glcm_Imc2 | T1 | Training | 0.342±0.115 | 0.275±0.093 | 0.665 | 0.004 | 0.908 |
|  |  | Internal Validation | 0.330±0.131 | 0.324±0.101 | 0.500 | 0.872 |  |
|  |  | External Validation | 0.376±0.119 | 0.284±0.068 | 0.734 | 0.044 |  |
| wavelet-LLH_firstorder_Median | T1 | Training | 2.076±10.930 | 8.181±7.790 | 0.634 | 0.004 | 0.894 |
|  |  | Internal Validation | 4.787±4.794 | 7.320±12.137 | 0.563 | 0.362 |  |
|  |  | External Validation | 4.459±9.660 | 2.749±4.854 | 0.552 | 0.619 |  |
| wavelet-HHL_glcm_Idmn | T2FS | Training | 0.935±0.028 | 0.921±0.021 | 0.673 | 0.014 | 0.957 |
|  |  | Internal Validation | 0.930±0.028 | 0.926±0.023 | 0.513 | 0.593 |  |
|  |  | External Validation | 0.965±0.027 | 0.963±0.030 | 0.510 | 0.909 |  |
| lbp-3D-k_glcm_Imc2 | T2FS | Training | 0.088±0.016 | 0.105±0.043 | 0.647 | 0.013 | 0.938 |
|  |  | Internal Validation | 0.093±0.019 | 0.106±0.033 | 0.640 | 0.120 |  |
|  |  | External Validation | 0.117±0.055 | 0.123±0.046 | 0.608 | 0.804 |  |
| wavelet-HLH_glcm_MCC | T2FS | Training | 0.395±0.058 | 0.361±0.055 | 0.678 | 0.005 | 0.972 |
|  |  | Internal Validation | 0.372±0.054 | 0.380±0.066 | 0.523 | 0.662 |  |
|  |  | External Validation | 0.421±0.083 | 0.463±0.143 | 0.566 | 0.406 |  |
